# SBMN: Similarity-Based Memory Network for the Diagnosis of Vertical Root Fracture in Dental Imaging

**DOI:** 10.3390/diagnostics16050710

**Published:** 2026-02-27

**Authors:** Jie Wang, Xin Yan Jin, Yi Fan Zhang, Jie Yuan, Zi Tong Lin, Ying Chen

**Affiliations:** 1 School of Electronic Science and Engineering, Nanjing University, Nanjing 210023, China; 2 Department of Dentomaxillofacial Radiology, Nanjing Stomatological Hospital, Medical School of Nanjing University, Nanjing 210008, China

**Keywords:** vertical root fracture, dental diagnostics, AI, CBCT, similarity-based memory network, category memory

## Abstract

**Background/Objectives:** Medical image analysis of vertical root fractures (VRFs) is challenged by limited annotated data, class imbalance, and subtle inter-class differences. To address these issues, we propose an SBMN: a Similarity-Based Memory Network that integrates Category Memory with the Basic SBMN Module and a similarity-based classifier. **Methods:** An SBMN stores representative features for each class and leverages similarity-based gating to enhance feature discrimination. Experiments were conducted on a CBCT dataset of fractured and non-fractured teeth to evaluate performance. **Results**: The SBMN achieved up to 97.1% and 99.7% classification accuracy on automatically and manually segmented images, respectively. Memory manipulation experiments confirm the critical role of Category Memory in controlling classification outcomes. **Conclusions:** These results indicate that SBMNs offer an effective and interpretable approach for small-sample medical image classification and diagnosis.

## 1. Introduction

Vertical root fracture (VRF) is a longitudinal fracture that begins at the apical root and extends coronally, often detectable on facial or lingual surfaces [1,2,3,4]. Classified by the American Association of Endodontists as the most severe type of cracked tooth, VRF accounts for 2–5% of tooth fractures [5] and ranks as the third leading cause of tooth loss [6]. It occurs in both endodontically and non-endodontically treated teeth, though the prevalence is considerably higher in the former [7,8,9]. Interestingly, most cases in untreated teeth have been reported in Chinese populations, likely reflecting dietary differences [10]. Despite its clinical significance, early diagnosis of VRF remains challenging because clinical manifestations are often subtle, nonspecific, or completely asymptomatic until substantial periapical destruction occurs. This delay frequently leads to misdiagnosis, inappropriate treatment, and eventual extraction [11,12]. Moreover, clinical presentations often mimic periodontal disease [13], and conventional diagnostic tools—including illumination, staining, bite tests, inspection, surgical exploration, and radiography—are limited in reliability [14]. Radiographs in particular may fail when the X-ray beam is not aligned with the fracture plane, leaving surgical inspection as the only definitive method, which is invasive and potentially damaging [15]. Collectively, these limitations not only compromise diagnostic accuracy but also increase clinical workload, highlighting the need for more reliable, reproducible, and non-invasive decision-support strategies.

Cone-beam computed tomography (CBCT), introduced in the late 1990s to reduce radiation exposure [16], has become a major advancement in dentomaxillofacial imaging and is widely applied to root canal visualization and VRF detection [17,18]. However, when fracture lines approximate the voxel size of CBCT, they may appear blurred, making diagnosis heavily dependent on radiologist expertise [16,19]. Consequently, although CBCT provides three-dimensional information, accurate interpretation still depends heavily on expert experience and careful slice-by-slice inspection. This manual review process is time-consuming in routine practice, particularly when screening large CBCT volumes, and may lead to inter-observer variability across clinicians. In recent years, artificial intelligence (AI) has increasingly been adopted as an assistive tool in medical image analysis. Convolutional neural networks (CNNs) remain dominant in computer vision and have achieved substantial success in diverse imaging tasks [20,21,22]. More recently, Transformer-based models [23], including Vision Transformer (ViT) [24] and Swin Transformer [25], have further improved image classification and segmentation performance. Nevertheless, these models typically require large-scale pretraining to generalize effectively, whereas VRF datasets—like many biomedical datasets—are small, noisy, and imbalanced. Such characteristics hinder stable clinical deployment of conventional networks and frequently result in overfitting or poor generalization across institutions. In this context, memory-augmented neural networks offer a promising solution. By incorporating an external memory module that can be selectively read from and written to during training and testing, these networks can retain task-relevant information and mitigate overfitting. This mechanism enhances generalization on small and heterogeneous datasets, making memory networks particularly suitable for biomedical applications such as VRF diagnosis, where data scarcity is a major challenge.

Additionally, our dataset was collected from a single center using a single CBCT device, which may limit generalizability. Despite this limitation, from a translational perspective, an AI-assisted VRF diagnostic system can be integrated into routine clinical workflows. In practice, CBCT volumes can first undergo automatic tooth segmentation, followed by per-tooth classification to generate fracture probability scores. These predictions can serve as a rapid screening or decision-support mechanism, highlighting suspicious regions for radiologists or endodontists, particularly in early-stage fractures, subtle fracture lines, or anatomically complex cases.

In this paper, we propose a novel memory network based on the calculation of similarity (SBMN) to classify VRF CBCT images. A key innovation of our approach is the introduction of category memory, which is stored independently of the network architecture and preserves the distinctive feature representations of each image category. Inspired by the attention mechanism in Transformer models [23], we design a similarity computation strategy that measures the relationship between stored category memory and features extracted from new input images. Within the SBMN module, category memory and input features are mutually updated: the stored memory guides the refinement of incoming features, while the new inputs simultaneously update and enrich the category memory. This bidirectional interaction enables more robust feature learning and enhances classification performance on small-scale medical datasets.

The contributions of our method can be summarized into three folds:

1. We propose a novel classification framework that integrates similarity-based computation with category memory, enabling automatic diagnosis of VRFs.
2. To ensure effective categorization of memory, we design a dedicated similarity loss function. By accurately aligning category memory with the corresponding class, the memory can better enhance the features extracted from input images, thereby improving classification—and ultimately diagnostic—accuracy.
3. We introduce a bidirectional update mechanism between category memory and input features during training. On the one hand, category memory is continuously refined by assimilating the characteristics of images from its corresponding class. On the other hand, each input image retrieves relevant memory components for fusion and feature enhancement, leading to more robust representation learning.

## 2. Related Work

### 2.1. Convolutional Neural Network (CNN)

Convolutional Neural Networks (CNNs) [26] have played a foundational role in computer vision, with their theoretical roots in the concept of the receptive field. In 1980, Fukushima introduced a neural architecture incorporating convolutional and pooling operations [27], which later inspired LeNet-5 [28], the first convolutional network successfully trained using backpropagation. Despite these early contributions, CNNs gained widespread recognition only after the success of AlexNet [21], which demonstrated the effectiveness of deep convolutional models on large-scale visual recognition tasks. This breakthrough accelerated the development of deeper and more structured architectures, including VGG [29], GoogLeNet [30], ResNet [22], DenseNet [31], and EfficientNet [32]. Recent surveys have systematically reviewed the evolution of CNN architectures and their applications in image classification, segmentation, and object detection, highlighting ongoing innovations in network design and efficiency [33,34,35]. Although Transformer-based models [23] have shown strong performance in various vision tasks, CNNs have not been supplanted but instead refined and modernized. Notably, ConvNeXt [36] demonstrates that well-designed CNNs can achieve performance comparable to, or even exceeding, that of vision transformers on multiple benchmarks. Motivated by these developments, we adopt CNNs as the backbone of the proposed SBMN framework to extract discriminative features from VRF CBCT images.

### 2.2. Transformer and Similarity

The emergence of the Transformer architecture [23] has profoundly impacted artificial intelligence, rapidly establishing itself as a leading framework for numerous natural language processing (NLP) tasks [37,38,39,40,41]. Beyond NLP, Transformers have also been successfully applied to core computer vision (CV) tasks, including segmentation [42,43,44,45], object detection [46,47,48], and image classification [24,25,49,50], as well as other vision-related applications. The key to their success lies in the self-attention mechanism, which captures correlations among embedded tokens. In NLP, higher attention weights reflect stronger semantic or contextual relationships. In vision, feature representations are more spatially grounded, and CNN-based architectures remain highly effective, particularly in extracting and hierarchically analyzing discriminative features for classification and segmentation. Recent surveys demonstrate that Transformers and their hybrid CNN–Transformer models continue to evolve across image and video tasks, addressing the trade-off between local and global dependencies [51,52,53,54]. These studies provide a systematic overview of Vision Transformers, their variants, and comparative analyses with CNNs, highlighting ongoing innovations in architecture design and task-specific adaptations.

### 2.3. Memory Networks

The concept of memory in neural networks [55,56] dates back to the late 20th century, with recurrent neural networks (RNNs) [57] and long short-term memory (LSTM) [58] designed to capture long-range dependencies in sequential data. However, the “memory” in these architectures is limited to internal hidden states, which are inherently transient and unstable over extended sequences. To address tasks requiring retention of earlier information—such as document-based question answering—memory networks with external memory modules were introduced [59,60,61]. In recent years, memory networks have gained increasing attention in computer vision, with applications in video object segmentation [62,63,64], domain adaptation [65], anomaly detection [66,67,68,69], and image classification [70]. Unlike conventional memory networks that store and retrieve past inputs as static memory, modern approaches leverage similarity-based interactions between inputs and stored memory. Recent advances in similarity-based and Hopfield memory mechanisms provide enhanced capacity and retrieval dynamics for vision tasks, including anomaly detection and spatiotemporal segmentation [71,72,73]. This design allows simultaneous refinement of both input features and memory content, enabling dynamic updates that improve overall network performance.

## 3. Materials and Methods

### 3.1. Materials

#### 3.1.1. Patients and Datasets

We used a dataset composed of 216 patients (126 males and 90 females, with an age range between 19–86 years). All CBCT images were collected by a NewTom VG scanner (QR SRL, Verona, Italy) with a voxel size of 0.15 mm, 110 kV, 3.6–3.7 mA, a field of view of 12 × 8 cm, and an acquisition time of 5.4 s. The selection criteria for CBCT images of VRF teeth were as follows: (a) non-endodontically treated teeth; (b) images exhibiting high quality and free from any artifacts, such as motion or beam hardening artifacts. For CBCT images of non-VRF teeth, three types of teeth were selected: (a) healthy teeth; (b) apical periodontitis teeth caused by caries; (c) periodontitis teeth with horizontal bone loss. Of all CBCT images from these patients, 139 VRF and 139 non-VRF teeth were independently annotated by two experienced radiologists, each with more than 10 years of clinical experience. Inter-examiner agreement between the two radiologists was almost perfect (Kappa = almost perfect), while intra-examiner agreement for each radiologist was also almost perfect. In addition, a third radiologist, blinded to the study design and patient information, performed a repeat evaluation three months later, showing substantial agreement with the consensus annotation (Kappa = 0.711). This procedure ensured high-quality and reliable labeling of VRF and non-VRF teeth. The study was approved by the Ethics Committee of Nanjing Stomatological Hospital, Medical School of Nanjing University [refEthics2018NL044], and the requirement for written informed consent was waived by the Ethics Committee. While the dataset is de-identified, we acknowledge that AI models may introduce biases, especially when applied to vulnerable populations or groups underrepresented in the dataset. Future work should carefully monitor potential biases and ensure equitable application of AI-based diagnostic tools [74].

#### 3.1.2. Image Preprocessing

The collected CBCT images were segmented using two methods: auto segmentation and manual segmentation. The auto segmentation method, consisting of five steps, was introduced by Hu et al. [75]. The effectiveness and reliability of this automatic tooth segmentation approach were independently validated in the same study, which reported accurate and stable performance on CBCT images. Furthermore, all automatically segmented results in our study were visually reviewed by experienced radiologists to ensure anatomical plausibility before further analysis. The manual segmentation method involved experienced radiologists manually segmenting VRF and non-VRF teeth from the same raw dentition images. Table 1 shows the specific segmentation results of our study. After segmentation, the segmented slices were resized to 224 × 224 to match the fixed input size required by the network, and gray-level transformation was applied to the images. Considering that this is a medical dataset with relatively small volume and potential biases, networks are prone to overfitting. Therefore, we applied several data augmentation methods. Specifically, the input images were randomly flipped horizontally and vertically, color jitter was applied (brightness = 0.5, contrast = 0.5, saturation = 0.5, hue = 0.3), and images were randomly rotated within the range of −90° to 90°. All augmentation methods were applied only to the training dataset, while all segmented images were normalized before being fed into the network.

### 3.2. Methods

In this section, we first provide a concise overview of our similarity-based memory network architecture for the classification and diagnosis of vertical root fractures (Section 3.2.1). We then describe each component of the network in detail (Section 3.2.2), followed by a comprehensive explanation of the associated loss functions (Section 3.2.3).

#### 3.2.1. Overview

**Figure 1:**
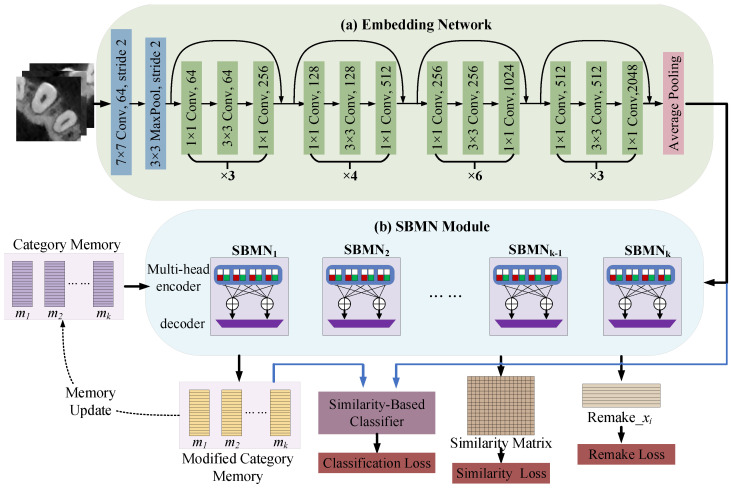
Overall framework of the Similarity-Based Memory Network (SBMN). The architecture consists of two main components: (**a**) an embedding network implemented with ResNet50, and (**b**) the SBMN module. Black arrows indicate the general data flow, while blue arrows represent the process in which the embedding features and the modified category memory are combined and fed into a similarity-based classifier to generate the final prediction.

The overall architecture of an SBMN is illustrated in Figure 1. The central component is the SBMN module, highlighted in light blue. This module takes two types of inputs: (i) the image feature representations extracted by the embedding network (implemented with ResNet50 in this study), and (ii) the category memory. It produces three outputs, namely *remake*_$x_{i}$, similarity matrix, and modified category memory. To generate the final classification result, we construct a similarity-based classifier using the embedding network output together with the modified category memory, replacing the conventional MLP classifier. The *remake*_$x_{i}$ and similarity matrix are used to compute the Remake Loss and Similarity Loss, respectively, while the modified category memory is iteratively fed back to replace the original memory for memory refinement. Within the SBMN module, multiple basic SBMN units are stacked to perform a sequence of operations—primarily similarity computation—on the two input branches, ultimately producing the aforementioned outputs. Additional architectural details are provided in the following subsections.

#### 3.2.2. Network Architecture

##### Embedding Network

The embedding network is responsible for extracting features from the input images. The SBMN module is designed as a universal architecture that can be coupled with various embedding networks, provided that their output dimensionality is fixed to *H* (e.g., H=2048, which is used by default). Both CNN-based architectures, such as ResNet and DenseNet, and Transformer-based architectures, such as ViT, can serve as embedding networks. All embedding networks are initialized with parameters pre-trained on ImageNet classification.

##### Category Memory

Category Memory (CM) is a memory collection that stores *k* sets of memory corresponding to each category, where $CM\in R^{M\times k\times H}$ refers to the size of each memory set (default = 16), *k* is the number of categories to be classified depending on the task, and *H* denotes the dimension of each slice of memory, which is set to be the same as the output dimension of the embedding network. Similarly to [76], our CM consists of feature representations of input images, which significantly reduces memory requirements. The CM is randomly initialized with mean 0 and variance 1. To address the issue of low consistency reported in [76], we apply a multi-head encoder to the memory before subsequent calculations, instead of using the raw memory directly. Furthermore, *m* can be set to a reasonable size, and the CM is updated after every training step.

##### Basic SBMN Module

**Figure 2:**
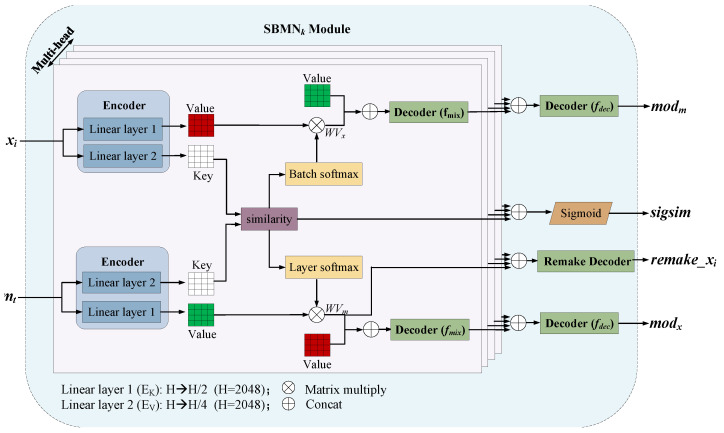
Schematic illustration of the basic SBMN module. Each Basic SBMN Module has a four-head design and independent parameters. Only one module is illustrated for clarity.

The main module, the SBMN Module, consists of *k* Basic SBMN Modules. Each Basic SBMN Module has the same structure but does not share parameters. The Basic SBMN Module has four heads structure, trying to capture different useful information. All heads within a Basic SBMN Module are independent. For clarity, only one representative Basic SBMN Module, including its multi-head structure, is shown in Figure 2.

**Encoder.** The encoder takes both feature representation and *t*-th category memory as inputs, where $t\in \left\{1,2,\ldots ,k\right\}$. We denote by $x_{i}\in R^{B\times H}$ and $m_{t}\in R^{M\times H}$ for feature representation and *t*-th category memory. Here, *B* denotes the batch size of the current input. The encoder consists of two different linear layers, $E_{K}$ and $E_{V}$ to generate Key and Value respectively, denoted by $K_{x}\in R^{B\times H/4}$, $V_{x}\in R^{B\times H/2}$, $K_{m}\in R^{M\times H/4}$ and $V_{m}\in R^{M\times H/2}$. We depict the process as follows.

(1) $$K_{x},K_{m}=E_{K}\left(x_{i},m_{t}\right)$$

(2) $$V_{x},V_{m}=E_{V}\left(x_{i},m_{t}\right)$$

**Similarity Calculation and Weighting.** After acquiring $K_{x}$, $K_{m}$, drawing on the idea of contrastive learning, we decide to determine the degree of modification of Category Memory and $x_{i}$ through the calculation of similarity. We can express this process with the following formula:

(3) $$sim=K_{x}⊙K_{m}^{T}$$

(4) $$W_{m}=layer\_softmax(sim)$$

(5) $$W_{x}=batch\_softmax(sim)$$

(6) $$WV_{m}=W_{m}⊙V_{m}$$

(7) $$WV_{x}=W_{x}^{T}⊙V_{x}$$

where ⊙ denotes the matrix multiplication and *T* means matrix transpose. We denote by $sim\in R^{B\times H}$ the similarity of $x_{i}$ and $m_{t}$. layer_softmax and batch_softmax are both softmax normalization operations, the only difference between them is that layer_softmax performs softmax on dimension *M*, while batch_softmax acts on dimension *B*. Then we calculate the weighted sum of $V_{m}$ and $V_{x}$ according to the normalized sim and denote them by $WV_{m}\in R^{B\times H/2}$ and $WV_{x}\in R^{M\times H/2}$. Before outputting the similarity vector $\bar{sim}\in R^{B\times 1}$, which measures how similar $x_{i}$ is to this certain category of memory $m_{t}$, sigmoid activation is required and then averaged on dimension *M*:

(8) sigsim=σ(sim)

(9) $$\bar{sim}=mean\left(sigsim\right)$$

here σ and mean() refers to the sigmoid function and the average operation on dimension *M*.

**Decoder.** We use calculated weighted sum $WV_{x}$ to modify $m_{t}$ and $WV_{m}$ to modify $x_{i}$. We concatenate $WV_{x}$ with $V_{M}$ and $WV_{m}$ with $V_{x}$, denoted by $\tilde{mod_{m}}$ and $\tilde{mod_{x}}$. Then we put four heads of $\tilde{mod_{m}}$ and $\tilde{mod_{x}}$ into decoder. The decoder consists of two parts. The first part is a mix module consisting of a two-layer MLP, functioning as mixing $\tilde{mod_{m}}$ or $\tilde{mod_{x}}$. We denote this module as $f_{mix}$. The second part is $f_{dec}$ to combine separated multi-heads together. The total process is shown as follows:

(10) $$mod_{x}=f_{dec}\left[f_{mix}\left(\tilde{mod_{x}^{1}}\right),f_{mix}\left(\tilde{mod_{x}^{2}}\right),f_{mix}\left(\tilde{mod_{x}^{3}}\right),f_{mix}\left(\tilde{mod_{x}^{4}}\right)\right]$$

(11) $$mod_{m}=f_{dec}\left[f_{mix}\left(\tilde{mod_{m}^{1}}\right),f_{mix}\left(\tilde{mod_{m}^{2}}\right),f_{mix}\left(\tilde{mod_{m}^{3}}\right),f_{mix}\left(\tilde{mod_{m}^{4}}\right)\right]$$

where $mod_{x}\in R^{B\times H}$, [∗,∗] means concatenate operation.

**Remake Decoder.** The remake decoder is designed to validate the effectiveness of the stored memory and to exploit it appropriately. In the similarity calculation and weighting stage, we assume that the input representation can extract relevant information from the memory bank, and expect this information to be meaningful and capable of reconstructing the input representation. To achieve this, the decoder concatenates the weighted sums of category memories across all heads and passes the result through a feed-forward layer to obtain $remake\_x_{i}$, formulated as follows:

(12) $$remake\_x_{i}=f_{re\_dec}\left[WV_{m}^{1},WV_{m}^{2},WV_{m}^{3},WV_{m}^{4}\right]$$

##### Similarity-Based Classifier

**Figure 3:**
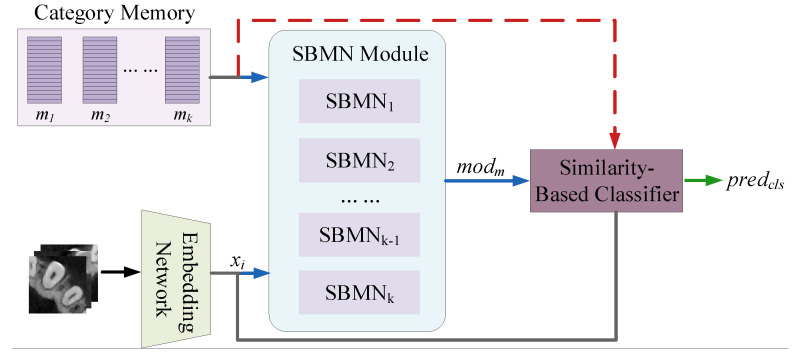
The c architecture of the similarity-based classifier. Different arrow styles and colors indicate the data flows: blue arrows represent the training data path, red dashed arrows indicate the testing data path, gray arrows denote shared inputs, and green arrows represent the output leading to the classifier.

To fully exploit the representational capability of the category memory within our network, we construct it as a classifier, as illustrated in Figure 3. Specifically, the model generates its final predictions by computing the similarity between the input feature representation and the category memory, with the resulting similarity scores serving directly as the classification output. Under this formulation, when the feature of an input sample is more similar to the memory of a particular class, the model naturally assigns a higher likelihood to that class.

A key detail is that the category memories used for similarity computation differ between the training and testing phases. During training, the model utilizes the class memories updated by the SBMN module. This enables the network to continuously refine and maintain an up-to-date and accurate memory bank through loss optimization and backpropagation. However, during testing, the category memories are kept unchanged because the updates generated by the SBMN module are no longer constrained by gradient optimization.

The similarity computation itself can be considered a gated similarity mechanism. Its formulation may either follow the same similarity-gating strategy used in the primary SBMN module or adopt a different similarity function. This flexibility allows the similarity-based classification module to be adapted to different datasets or specific application needs.

#### 3.2.3. Training Loss Functions

Here the overall training loss is a weighted sum of three different losses:

(13) $$L_{total}=\lambda_{cls}L_{cls}+\lambda_{sim}L_{sim}+\lambda_{remake}L_{remake}$$

where we denote by $L_{cls}$, $L_{sim}$ and $L_{remake}$ classification, similarity and remake losses, respectively. λ is weighted factor ranged between 0 to 1. The classification loss ensures the accuracy of classification to achieve the purpose of diagnose VRFs, the similarity loss helps maintain the validity of each set of memory in Category Memory, while remake loss allows us to generate feature representations of a certain category from stored Category Memory. The detailed descriptions of each loss are presented in the following.

**Classification Loss.** The final classification is performed using a similarity-based classifier. Given an input image, the embedding network produces a feature representation $x_{i}$, which is further processed by the SBMN Module to obtain the modified category memory $mod_{m}\in R^{M\times KH}$. The feature representation $x_{i}$ and the modified category memory $mod_{m}$ are jointly fed into the classifier to generate the final prediction:

(14) $$pred_{cls}=f_{cls}\left(x_{i},mod_{m}\right),$$

where $f_{cls}$ denotes the classifier and $pred_{cls}\in R^{B\times K}$ represents the predicted label probabilities of the input images. We employ the cross-entropy loss function to compute the classification loss:

(15) $$L_{cls}=-\sum_{i=1}^{K}label^{i}\log\left(pred_{cls}^{i}\right),$$

where $label^{i}=1$ if the input sample belongs to the *i*-th category, and $label^{i}=0$ otherwise.

**Similarity Loss.** The similarity loss is designed to help maintain the validity of Category Memory. As we all know, all inputs will modify Category Memory. The degree of modification depends on the similarity between their feature representations and a certain category of memory. The more similar it will be, the more it will be modified. To reduce the excessive memory modification resulting in one category of memory being modified to another, we design and use similarity loss function to restrict this. The loss function is shown below:

(16) $$L_{sim}=-\frac{1}{K}\sum_{i=1}^{K}\sum_{j}^{N}\left[label_{j}^{i}\;log\left({\bar{sim}}_{j}^{i}\right)+\left(1-label_{j}^{i}\right)log\left(1-{\bar{sim}}_{j}^{i}\right)\right]$$

where *N* denotes the total num of inputs and the label here is in one-hot form.

**Remake Loss.** The remake loss is originally designed to explore the use of memory. We wish that under some certain kinds of stimulus, typical feature representations of a certain category can be restored from memory. Here we use $remake\_x_{i}$, denoted as $rx_{i}$, together with $x_{i}$ to calculate remake loss.

(17) $$L_{remake}=MSE\left(rx_{i},x_{i}\right)={∥rx_{i}-x_{i}∥}_{2}^{2}$$

## 4. Results

We choose to use SGD as our optimizer. The SGD weight decay is set as 0.0004 and the SGD momentum is 0.9. We use a mini-batch size of 32 and an initial learning rate of 0.005. The learning rate adjustment strategy is to multiply the current learning rate by 0.95 after every 5 epochs, the minimum learning rate is 0.00001. We train our model on Tesla V100 GPU with 16G memory. These hyperparameters were selected based on prior studies in small-sample medical imaging tasks and preliminary experiments on our dataset, which indicated stable convergence and high classification performance.

### 4.1. Evaluation Metrics

In order to evaluate the performance of our model quantitatively, we chose to calculate diagnostic accuracy, sensitivity, specificity, positive predictive value (PPV) and F1 score of both auto-segmentation dataset and manual segmentation dataset. Since we only need to distinguish the input images with or without fracture, we can easily have access to the confusion matrix of the classification results, then we can calculate the metrics mentioned above. In addition, the random division of datasets may bring contingency and uncertainty to the classification results, thus causing errors in the evaluation of network performance. To avoid this condition, we conducted five-folds cross validation and calculated the average metrics as final results.

### 4.2. Results of SBMN

An SBMN exhibits strong flexibility and can be readily adjusted by employing various embedding networks. Considering that automatic segmentation better reflects realistic clinical workflows, the auto-segmentation results are regarded as the primary evaluation, whereas manual segmentation is used only as a controlled reference. In this study, we empirically experiment with VGG19, ResNet50, DenseNet169, ResNeXt, and ViT-B as embedding networks of SBMNs for binary classification. The corresponding results are presented in Table 2.

As shown in Table 2, within the auto-segmentation group, the ResNet50-based SBMN achieves the highest accuracy and F1 score, whereas the ResNeXt-based SBMN attains the best PPV and specificity. Specifically, the ResNet50-based SBMN reaches an accuracy of 97.1%, a sensitivity of 98.6%, and an F1 score of 97.1%, which are 2.5%, 4.9%, and 2.6% higher than the second-best results, respectively. Meanwhile, the ResNeXt-based SBMN achieves the highest specificity (97.1%) and PPV (97.0%), outperforming the ResNet50-based model by 1.4% and 1.2%. In contrast, the Inception_v3-based SBMN shows inferior performance compared with other backbones, while the remaining networks maintain similar and relatively high results.

In the manual-segmentation group, all models exhibit higher performance overall. This improvement is expected because experienced radiologists manually exclude low-quality or ambiguous regions, which reduces background interference and may lead to performance inflation. Therefore, these results should be interpreted as an upper-bound reference under ideal conditions rather than a realistic clinical scenario. Since clinical diagnosis prioritizes the detection of all potential vertical root fractures, sensitivity remains an important metric. The ResNet50-, VGG19-, ResNeXt-, and ViT-B-based SBMNs all reach 100.0% sensitivity; however, this may reflect the specific characteristics and limited size of our datasets rather than definitive model superiority. The DenseNet169-based SBMN achieves the highest specificity and PPV. Overall, the ViT-based SBMN demonstrates the highest accuracy and F1 score (99.7%), but these results should be interpreted with caution given the sample size and dataset characteristics. Considering the limited sample size, only descriptive statistics including accuracy, sensitivity, specificity, PPV, and F1 score are reported. Inferential statistics such as confidence intervals or *p*-values were not calculated, as they may not be reliable with the current dataset. Future studies with larger sample sizes will allow more robust statistical analyses.

### 4.3. Comparisons with Other Networks

To further validate the effectiveness of the proposed model, the ResNet50-based SBMN is compared with several representative convolutional neural networks (CNNs), including VGG19, ResNet50, DenseNet169, Inception_v3, and ResNeXt, as well as Transformer-based architectures such as ViT-B, Swin-T, and Mobile-ViT, alongside the SCCNN model. For fair comparison, the compared models are equipped with multilayer perceptron (MLP) classifiers of identical input dimensions to minimize the potential influence of parameter quantity on the evaluation metrics. In addition, radiologists are invited to diagnose the manually segmented dataset to provide reference metrics. The comparative results are summarized in Table 3.

As shown in Table 3, the proposed SBMN consistently outperforms all other networks across most evaluation metrics. In the auto-segmentation group, the SBMN achieves the highest accuracy 97.1% and sensitivity 98.6%, which are 3.2% and 3.6% higher than the second-best model, respectively. In the manual-segmentation group, the SBMN ranks first in accuracy, sensitivity, and F1 score, while its specificity and PPV remain within 1.4% of the highest results. Furthermore, stable performance under automatic segmentation demonstrates the feasibility of deep learning-based assistance in realistic clinical scenarios.

### 4.4. Visualization

To gain a more intuitive understanding of the Category Memory mechanism and the effect of the SBMN module on the feature extraction capability of the embedding network, several visualization experiments are conducted to analyze its influence on the feature representations. Specifically, two images are randomly selected from the datasets with vertical root fractures (VRF) and without fractures (non-VRF), forming three comparison groups: (a) non-VRF vs. non-VRF, (b) non-VRF vs. VRF, and (c) VRF vs. VRF.

**Figure 4:**
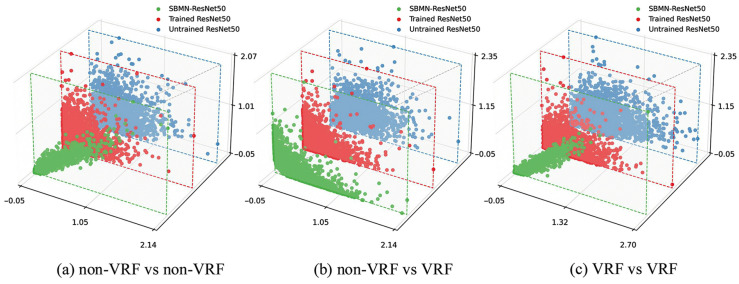
Visualization of feature distributions under different network configurations. (**a**) non-VRF vs. non-VRF, (**b**) non-VRF vs. VRF, and (**c**) VRF vs. VRF. Three models are compared: Untrained ResNet50, Trained ResNet50, and the SBMN (based on the ResNet50 embedding network). Each point represents one feature channel, where the horizontal and vertical coordinates indicate the corresponding feature values from two images.

As illustrated in Figure 4, three-dimensional scatter plots are generated to demonstrate the feature distributions under different network configurations, corresponding to the Untrained ResNet50, Trained ResNet50, and SBMN (based on the ResNet50 embedding network) models. After feeding each image pair into the networks, the embedding feature representations are obtained as $x_{i}\in R^{H}$ (where H=2048). In the 3D scatter plots, each point represents one feature channel, with the horizontal and vertical axes indicating the feature values of the same channel from the two images, respectively. These visualizations intuitively reveal how the introduction of the SBMN module influences the structure and discriminative characteristics of the feature distributions.

In the untrained ResNet50, the scatter points are widely dispersed and lack clear patterns, indicating that the extracted features across channels exhibit weak discriminative consistency. After training on the tooth fracture dataset, the ResNet50 shows more organized feature distributions: the scatter points of same-class image pairs (non-VRF–non-VRF or VRF–VRF) are distributed closer to the 45° diagonal line, reflecting higher inter-channel similarity, whereas those of different-class pairs (non-VRF–VRF) are located nearer to the coordinate axes, suggesting greater inter-class differences. When the SBMN is employed, these differences become even more pronounced. Scatter points for same-class pairs are densely distributed along and around the 45° diagonal line, while those for different-class pairs are mainly concentrated near the coordinate axes. This observation indicates that the SBMN module significantly enhances the embedding network’s discriminative feature extraction capability, enabling it to more effectively distinguish between intra-class and inter-class inputs, thereby improving the overall feature representation performance.

**Figure 5:**
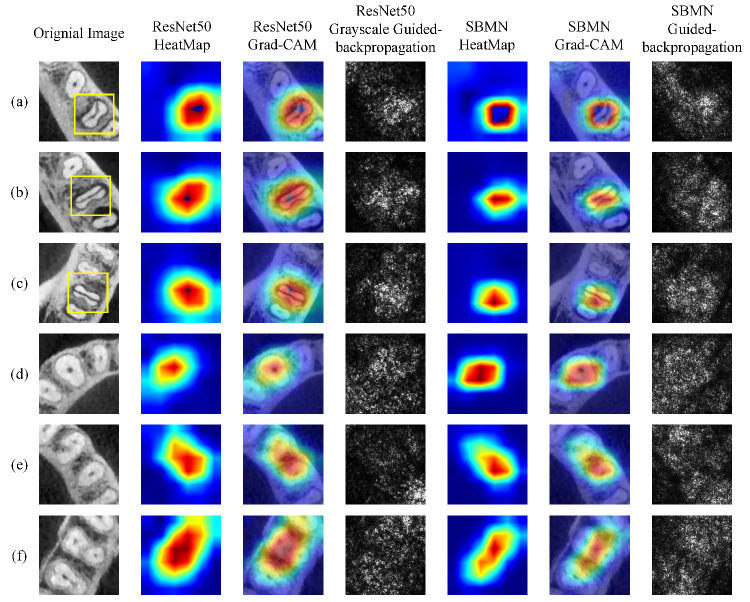
Visualization comparison between ResNet50 and the SBMN using Grad-CAM and guided backpropagation methods. Columns from left to right show: original CBCT images, ResNet50 heatmaps, ResNet50 Grad-CAM, ResNet50 grayscale guided backpropagation, SBMN heatmaps, SBMN Grad-CAM, and SBMN guided backpropagation. Rows (**a**–**c**) correspond to VRF teeth, and rows (**d**–**f**) correspond to non-VRF teeth. Yellow boxes mark the fracture regions in VRF cases.

To further investigate how the SBMN module affects the network’s focus on diagnostically relevant regions, we employ visualization techniques to compare feature localization between ResNet50 and the SBMN (based on the ResNet50 embedding network). Figure 5 presents the results obtained using Grad-CAM and guided backpropagation. Three representative CBCT images with vertical root fractures (VRF) and three non-VRF images are selected for visualization. Figure 5a–c depict fractured teeth, where yellow rectangles indicate the specific locations of root fractures, while Figure 5d–f show non-fractured teeth.

As illustrated, ResNet50 exhibits relatively diffuse activation regions that are not precisely aligned with the actual fracture sites. In contrast, the SBMN produces heatmaps and activation distributions that are more concentrated and better correspond to the clinically relevant fracture areas. This observation indicates that the incorporation of Category Memory enhances the network’s attention to diagnostically important regions, thereby improving interpretability and diagnostic reliability. Moreover, the guided backpropagation results of the SBMN also reveal finer texture details within the fracture zones, further confirming its superior capability in identifying subtle structural variations.

### 4.5. Ablation Study

To further investigate the effect of different similarity calculation strategies within the proposed SBMN framework, four commonly used methods are compared: dot product, cosine similarity, Pearson correlation, and Euclidean distance. Table 4 summarizes the evaluation metrics obtained from each approach, including accuracy, sensitivity, specificity, PPV, and F1 score.

Among all tested methods, the dot product achieves the best overall performance, yielding the highest accuracy (97.1%) and F1 score (97.1%), as well as superior sensitivity (98.6%). This result suggests that the dot product provides a more effective measure of feature consistency in the latent representation space, facilitating more discriminative learning for vertical root fracture (VRF) detection. Cosine similarity shows comparable results, with slightly higher specificity (98.2%) and PPV (96.4%), indicating its robustness in distinguishing negative samples. In contrast, both Pearson correlation and Euclidean distance perform relatively worse across all metrics, implying that linear correlation and distance-based similarity are less capable of capturing subtle semantic relationships in feature embeddings. In conclusion, the results demonstrate that different similarity-gated computation methods can all be effectively integrated within the SBMN framework. Depending on specific application scenarios and computational requirements, the similarity function can be flexibly adjusted to achieve an optimal balance between discriminative performance and efficiency.

## 5. Discussion

This study evaluated the clinical feasibility and diagnostic performance of the proposed Similarity-Based Gated Memory Network (SBMN) for detecting vertical root fractures (VRFs) on CBCT images using a relatively small real-world dataset. The primary aim was to improve diagnostic reliability under small-sample conditions by incorporating a memory mechanism that stabilizes feature learning and reduces overfitting. The main contributions and findings of this work can be summarized as follows:

1. **Introduction of Category Memory:** We introduce a category-level memory mechanism that preserves representative patterns of fractured and non-fractured teeth. By retaining stable class-specific information across training samples, the model can better handle limited and heterogeneous medical data, which are common in clinical studies.
2. **Design of the Basic SBMN Module:** This similarity-guided design enables the network to selectively emphasize clinically relevant features while suppressing noise and irrelevant structures frequently observed in CBCT images. By integrating similarity computation with gating operations and a similarity loss function, this module ensures the stability of the Category Memory repository while maintaining discriminative and robust feature representations.
3. **Proposal of the Similarity-based Classifier:** Instead of relying solely on conventional fully connected classifiers, predictions are generated based on similarity between current inputs and stored category representations. This strategy provides a more transparent decision process and improves interpretability, which is desirable for clinical adoption.
4. **Experimental Validation on Small Medical Datasets:** On a small dataset of vertical root fractures (VRF), the SBMN achieved high diagnostic accuracy on both automatically and manually segmented images, indicating that the memory mechanism can effectively capture subtle structural differences associated with fractures. Importantly, competitive performance was maintained under automatic segmentation, which better reflects realistic clinical scenarios where manual annotation is not feasible.

The proposed framework can be seamlessly integrated into routine clinical workflows through automatic tooth segmentation and subsequent tooth-level fracture probability estimation, thereby serving as a rapid screening or decision-support tool rather than replacing expert judgment. Misclassified cases predominantly involved teeth with very thin fracture lines, severe imaging artifacts, or complex periodontal destruction. Although analysis of these errors provides a general understanding of clinically challenging scenarios, the specific features underlying the network’s decisions remain inherently opaque. Accordingly, such error analysis should be regarded as a high-level descriptive assessment rather than a definitive explanation of the model’s decision-making process. This study is limited by the use of a single-center dataset acquired from a single CBCT device, which may restrict generalizability. In addition, the relatively limited sample size may reduce statistical power, requiring larger cohorts to more reliably confirm the stability and reproducibility of the reported performance. While cross-validation demonstrates stable results and supports the robustness of the category memory mechanism across folds, the risk of overfitting cannot be entirely excluded. In practical applications, additional training or fine-tuning may be necessary for different CBCT scanners, populations, or imaging protocols to ensure consistent performance. Future multi-center and prospective studies are therefore warranted to further validate the robustness and generalizability of the proposed approach.

Overall, the results suggest that the SBMN is a promising decision-support approach for VRF detection, although further large-scale and multi-center validation is required before routine clinical deployment.

To further validate the effectiveness of the memory mechanism, we conduct experiments to analyze the relationships between the hidden Category Memory and the feature representations, both obtained through the encoder within the SBMN module. The experiments focus on the channel-level interactions between these two representations, aiming to demonstrate how Category Memory participates in and strengthens the feature extraction process of the embedding network.

**Figure 6:**
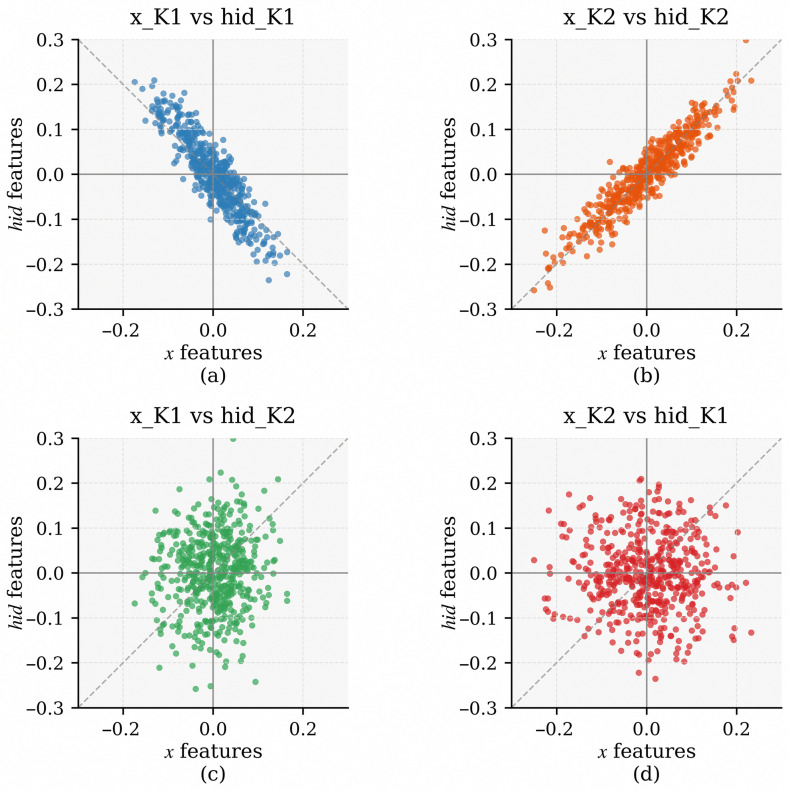
Channel-wise scatter distributions between the image features (x_K) and hidden Category Memory (hid_K) obtained from the encoder within the SBMN module for a randomly selected non-VRF CBCT image. Notations 1 and 2 indicate two different Basic SBMN modules. Panels (**a**,**b**) show intra-class and inter-class correlations within the same module, while panels (**c**,**d**) illustrate inter-module decorrelation.

As illustrated in Figure 6, we select one randomly chosen non-VRF image and plot the scatter distributions of 2048 channel pairs, where the *x*-axis represents the feature value from the image representation x_K, while the *y*-axis denotes the corresponding value from the memory representation hid_K, both obtained through the encoder within the SBMN module. Here, the notations 1 and 2 indicate that the representations are derived from two different Basic SBMN modules.

In Figure 6a, where the image feature and the memory representation belong to different classes, the scatter points are roughly distributed along the inverse diagonal Y=−X, indicating weak or negative correlation. In contrast, Figure 6b shows the case where the image and memory belong to the same class. Here, the scatter points align closely along the diagonal Y=X, reflecting strong positive correlation. This pattern is consistent with our expectations: the similarity-based gating computation in the SBMN performs a dot product between corresponding feature and memory vectors across all channels. Points along Y=X yield a large positive contribution to the similarity score, whereas points along Y=−X produce a small negative contribution. These results demonstrate that the current similarity computation effectively captures the relationship between feature representations and their corresponding Category Memory.

Furthermore, Figure 6c,d illustrate the relationships between two different Basic SBMN modules. The scatter points in these plots are randomly distributed around the origin, showing no apparent linear correlation. This inter-module decorrelation confirms that different SBMN modules independently encode distinct category-specific knowledge, avoiding feature redundancy and enhancing the overall discriminative capacity and interpretability of the network.

To further validate the effectiveness of the Category Memory, we perform memory manipulation experiments in which the stored memories are modified in various ways, including replacing all memories with zero vectors, using a single-class memory, random initialization, or swapping memories between different classes. These modified memories are then used as input to the network for classification prediction. The results show that when memories are replaced with zero vectors or a single-class memory, inter-class similarity scores become indistinguishable. Under these conditions, the network defaults to selecting the first indexed class, resulting in perfect accuracy for one class while completely misclassifying the other. In the randomized memory experiments, prediction performance fluctuates significantly, with most trials exhibiting a substantial drop in accuracy and only occasional instances of relatively high accuracy. In the memory swapping experiments, correct classification is achieved only when each memory unit contains the corresponding class-specific memory.

Collectively, these findings demonstrate that the network’s classification performance critically depends on the integrity and class-specific content of the Category Memory. Moreover, they confirm that modifying the memory can directly influence the network’s classification behavior, highlighting the central role of Category Memory within the SBMN framework.

## 6. Conclusions

To improve the classification and diagnosis of vertical root fractures (VRFs), we propose a novel Similarity-Based Memory Network (SBMN) that leverages Category Memory and the Basic SBMN Module to enhance feature representation and classification. The network stores representative features for each class in a structured memory, while the Basic SBMN Module regulates the interaction between input features and memory, ensuring stability and effective feature enhancement. Experiments on a small-scale CBCT dataset indicate that SBMNs achieve improved classification accuracy and feature discriminability compared with conventional networks. Channel-level analyses show that intra-class features align closely with their corresponding memory, whereas inter-class features are decorrelated, demonstrating that the network effectively captures class-specific information. Memory manipulation experiments further confirm the critical role of Category Memory, as classification performance depends on the integrity and specificity of the stored memories. Overall, SBMNs provide a robust framework for small-sample medical image analysis, delivering reliable diagnostic performance, enhanced interpretability, and consistent class-specific feature representation. Incorporating structured memory with the Basic SBMN Module offers an effective strategy for automated diagnosis in scenarios with limited data. However, given the current single-center dataset and lack of extensive multi-center validation, these findings primarily demonstrate methodological potential rather than immediate clinical readiness. Future studies with diverse scanners and populations are needed to confirm clinical applicability.

## Figures and Tables

**Table 1:** Dataset distribution for different segmentation methods and categories (number of teeth).

Segmentation	Category	Total (Train/Test)
Auto Segmentation	VRF	555 (416/139)
	Non-VRF	563 (424/139)
Manual Segmentation	VRF	276 (208/68)
	Non-VRF	276 (208/68)

**Table 2:** Comparison of SBMN with different backbones using auto and manual segmentation.

**Auto Segmentation**	**Accuracy**	**Sensitivity**	**Specificity**	**PPV**	**F1 Score**
SBMN (ResNet50)	97.1	98.6	95.7	95.8	97.1
SBMN (DenseNet169)	93.5	92.1	95.0	94.8	93.4
SBMN (VGG19)	94.6	93.5	95.7	95.6	94.5
SBMN (Inception_v3)	78.9	91.4	66.2	73.0	81.2
SBMN (ResNeXt)	94.6	92.1	97.1	97.0	94.5
SBMN (ViT)	92.4	93.7	91.2	91.2	92.4
**Manual Segmentation**	**Accuracy**	**Sensitivity**	**Specificity**	**PPV**	**F1 Score**
SBMN (ResNet50)	99.1	100.0	97.1	97.1	98.5
SBMN (DenseNet169)	98.4	97.1	100.0	100.0	98.5
SBMN (VGG19)	98.5	100.0	97.1	97.1	98.6
SBMN (Inception_v3)	80.1	80.9	79.4	79.7	80.3
SBMN (ResNeXt)	97.1	100.0	94.1	94.4	97.1
SBMN (ViT)	99.7	100.0	99.5	99.8	99.7

**Table 3:** Performance comparison of the SBMN and other representative CNN- and Transformer-based networks using auto and manual segmentation.

**Auto** **Segmentation**	**Accuracy**	**Sensitivity**	**Specificity**	**PPV**	**F1 Score**
ResNet50	91.4	92.1	90.7	90.8	91.4
DenseNet169	87.1	80.6	93.5	92.6	86.6
VGG19	87.8	89.2	86.3	86.7	87.7
Inception_v3	87.1	84.9	89.2	88.7	86.8
ResNeXt	92.4	92.8	92.1	92.1	92.5
SCCNN	93.9	95.0	92.9	93.0	94.0
ViT-B	90.6	90.1	91.1	90.9	90.6
Swin-T	91.5	92.8	90.2	90.4	91.5
Mobile-ViT	87.9	86.5	89.3	88.9	87.9
SBMN (ResNet50)	97.1	98.6	95.7	95.8	97.1
**Manual** **Segmentation**	**Accuracy**	**Sensitivity**	**Specificity**	**PPV**	**F1 Score**
ResNet50	97.8	97.0	98.5	98.5	97.8
DenseNet169	96.3	94.1	98.5	98.5	96.2
VGG19	94.9	92.7	97.0	96.9	94.8
Inception_v3	87.8	86.3	89.2	88.9	87.6
ResNeXt	97.8	97.1	98.5	98.5	97.8
SCCNN	98.0	98.3	96.2	97.8	97.2
ViT-B	98.2	98.1	98.3	98.2	98.2
Swin-T	98.6	98.4	98.2	98.3	98.3
Mobile-ViT	97.4	96.4	98.2	98.1	97.3
SBMN (ResNet50)	99.1	100.0	97.1	97.1	98.5

**Table 4:** Evaluation metrics of different similarity calculation methods.

Similarity Calculation Method	Accuracy	Sensitivity	Specificity	PPV	F1 Score
Dot Product	97.1	98.6	95.7	95.8	97.1
Cosine Similarity	97.0	97.6	98.2	96.4	97.0
Pearson Correlation	96.8	96.5	97.1	96.7	96.6
Euclidean Distance	94.7	94.9	94.6	95.1	95.0

## Data Availability

The data that support the findings of this study are not publicly available due to privacy and ethical restrictions. Data may be available from the corresponding author upon reasonable request.
